# Meteorological Observations

**Published:** 1867-02

**Authors:** J. G. Westmoreland

**Affiliations:** Atlanta, Ga.


					﻿I	** Z 02 z’odZ'* * xm'ZZ^Z^^^ZZ^^aiZZZZaa^
s
o
c' ------------------------------------------------------------------
4=2	§ S . . .HV^ > .«
I C>	55	C SZH P- . . .co .02 co .	. .	...	.... cc
?A<*-	C ’-'	m ZZZ Z 02 ooZcriZZcdtziZZZcdoQZZZZai
'S i •*
I ---------------------•---------------------------------------
Vi . e- I
e	.	..................
32	a • • .k’^^ • a a
£	<	8 Z S . Z02CC.O2.CQ	MZoi
1	o	“02Z	«	« odZZZZZ Z Z od Z Z Z-Z'02 02
e“®
►S =- '
-g
©J	”	©©o©©©o©©©©©©©o©©©©©©©©©©©o©©o©
.	TH rH r-^r^nH	rH Hr-lrf
*K> CZ2
O to-----------------------------------------------------------------
«• ® I §
,s	i	£7	S	©© ©© © ©© © ©©©©©©© ©o o© o o ©© © ©©©©©© ©
S'. |	§	«	-	~ —	-
-S I
Ci= -
'e °	s
c: >	©©©©©©©©©©©o©©©©©©©©©©©©©©©s©©©
« g	rH w	-r-P	W rt ,-i rH rirl
£ £
1	z________________________________________________________________
3i •	.	^	**	X
Zo£ 2	££	S3	g	g
M <zo §	' ’	• •	a
CS M K< Z s C4«	M «	02	M
02
*S	—
g S 3	—.-------------------------------------------------------
§	5	33a£3338SS33!3S33333W3m33S8SSSS
2	§ 2 —------------- -.. ..........................................
'<	«© o O M- O — to eO 00 CO -f '■f >O O) CO CO Oi <& O O Tf Ci to CO O ’f o O CO ”* »o
£-<	-< CO CO GO CO CO CO’t 00 CO CO X CO CO CO CO CO CO CO CO CO OJ CO CO Qi CO CO 4 iQ
§ ■g__________co_____________________________________________________
§s	IS *	°L	5	^7	°|T	°1
5	’ o_ gggggg?igggg§?[ggSgg§S§gggSga^^
S CO Ci GO CO CO CO CO <O tO-rf tO °£ CO b-t-^l’ CO <7D	CO	tO'O °7 to
•S	® °- °. °- °. °. °- °- • °. • °- °- °.	w o d 4 4 4 4 o o -2 o
?4	O	ct-cicicieicicicioicicicic>ciciciciciciciaioiciaicicicicioicici
Z»	'>4Cq04^010JOlOlC'l'MC^010JOJ01->?0^-»/>lCN01ClOJ0404Q40Ja<lCM->10J
X,	<4	*3 CO Oi CD CD CD CO I— W0 "l -+ tO -f< co b- b- CO CO °|’ CO 4 Tf '| t— CO tO t-
3^	•< C> ci ci o> ci ai oi C* ci ci ci C5 ©i CO ci ci ci C; c> ci C> ci oi O as ci o’ ci o> ci oi
~	______ to d0K0JC^0104^0404C<<0101Q4010>^0404040<C40J0ICJC4a‘JOCqQ4040104
				

